# First study on virulence genes, antimicrobial resistance, and integrons in *Escherichia coli* isolated from cage, free-range, and organic commercial eggs in Phayao Province, Thailand

**DOI:** 10.14202/vetworld.2022.2293-2301

**Published:** 2022-09-25

**Authors:** Achiraya Siriphap, Chittakun Suwancharoen, Watchara Laenoi, Parinya Kraivuttinun, Orasa Suthienkul, Watsawan Prapasawat

**Affiliations:** 1 Division of Microbiology and Parasitology, School of Medical Sciences, University of Phayao, Phayao 56000, Thailand; 2 Division of Animal Science, School of Agriculture and Natural Resources, University of Phayao, Phayao 56000, Thailand; 3 Program in Environment, Faculty of Science and Technology, Uttaradit Rajabhat University, Uttaradit 53000, Thailand; 4 Faculty of Public Health, Mahidol University, Bangkok 10400, Thailand; 5 Department of Clinic, Faculty of Veterinary Medicine, Mahanakorn University of Technology, Bangkok 10530, Thailand

**Keywords:** antimicrobial resistance, eggshells, *Escherichia coli*, integrons, virulence

## Abstract

**Background and Aim:**

Antimicrobial resistance (AMR) is a global problem that affects human and animal health, and eggs can act as a vehicle for pathogenic and non-pathogenic resistant bacteria in the food chain. *Escherichia coli* is an indicator of food contamination with fecal materials as well as the occurrence and levels of AMR. This study aimed to investigate the presence of AMR, integrons, and virulence genes in *E. coli* isolated from eggshell samples of three egg production systems, from supermarkets in Thailand.

**Materials and Methods:**

A total of 750 hen’s egg samples were purchased from supermarkets in Phayao Province: Cage eggs (250), free-range eggs (250), and organic eggs (250). Each sample was soaked in buffered peptone water (BPW), and the BPW samples were incubated at 37°C for 18–24 h. All samples were tested for *E. coli* by the standard conventional culture method. Then, all identified *E. coli* were tested for antimicrobial susceptibility to 15 antimicrobial agents by the agar disk diffusion method. All *E. coli* strains were subsequently found to have virulence genes and Classes 1 and 2 integrons by polymerase chain reaction.

**Results:**

Among the eggshell samples, 91 samples were identified as having *E. coli* (cage eggs, 24 strains; free-range eggs, 27 strains; and organic eggs, 40 strains). Then, among the *E. coli* strains, 47 (51.6%) were positive for at least one virulence gene. The proportion of AMR in the eggshell samples was 91.2% (83/91), and streptomycin (STR), ampicillin (AMP), and tetracycline (TET) had a high degree of resistance. Among the *E. coli* strains, 27 (29.7%) strains were positive for class 1 or 2 integrons, and integron-positive strains were commonly found in STR-, AMP-, and TET-resistant strains. Multidrug resistance (MDR) was detected in 57.1% (52/91) of the *E. coli* strains, with STR-AMP-TET (5.5%) as the most frequent pattern. The proportion of MDR in cage eggs was 75.0% (18/24), which was higher than in both free-range and organic eggs. On the other hand, 53.2% (25/47) of *E. coli* carrying virulence genes had MDR, distributed across the production systems as follows: Cage eggs, 76.9% (10/13); free-range eggs, 63.6% (7/11); and organic eggs, 34.8% (8/23).

**Conclusion:**

*Escherichia coli* was detected in eggshell samples from all three egg production systems. The high level of virulence genes, AMR, and integrons indicated the possibility of dissemination of AMR among pathogenic and commensal *E. coli* through eggshells. These findings could be a major concern to farmers, food handlers, and consumers, especially regarding raw egg consumption.

## Introduction

Eggs are the most commonly consumed food globally. They contribute significantly to a daily healthy diet containing protein with high nutrient values for consumption [1, 2]. In Thailand, egg production reaches about 14,810 million eggs per year, and the office of agricultural economics has the campaign to encourage the consumption of eggs to the annual goal of 300 eggs per person in 2023 [3]. However, in addition to offering health benefits, eggs may act as a vehicle for pathogenic resistance-bacterial contamination in the food chain [4]. In egg production industries, there are many housing systems for hen’s egg production that produces several types of eggs sold in markets: For example, cage eggs, free-range eggs, and organic eggs. At present, the risk of bacterial contamination, including *Escherichia coli* contamination, in each type of egg production is unknown.

*Escherichia col*i are the normal flora in intestinal tracts of healthy warm-blooded animals and humans, but some strains have evolved pathogenic mechanisms to cause disease [5]. Specifically, six pathotypes of *E. coli* can cause intestinal disease: Enteropathogenic *E. coli* (EPEC), enterohemorrhagic *E. coli* (EHEC), enterotoxigenic *E. coli* (ETEC), enteroaggregative *E. coli* (EAEC), enteroinvasive *E. coli*, and diffusely adherent *E. coli* [6]. Among bacteria found on eggshells, *E. coli* has been claimed to contaminate eggs through close contact areas and environments. Most *E. coli* on eggshells were originally in close contact with feces or had indirect contact from reused egg trays [7]. Furthermore, *E. coli* contamination on eggshells can be indicative of poor hygiene and sanitation on the farm [8]. However, *E. coli* contamination on eggshells derived from the three types of egg products (i.e., cage, free-range, and organic eggs) has not been investigated in Thailand.

Antimicrobial resistance (AMR) is increasingly recognized as a serious public health problem [9]. The overuse and misuse of antimicrobials on animal farms have encouraged AMR among bacteria in animal habitats [10]. Further, the possibility of increased transmission of antimicrobial-resistant bacteria to humans through the food chain has become a major concern in recent years [11]. In addition, the dissemination of antimicrobial-resistant genes by horizontal gene transfer has led to the rapid emergence of AMR among bacteria [12]. Integrons have been shown to play an important role in the evolution and dissemination of multidrug resistance (MDR) in Gram-negative bacteria [13]. There are three main classes of integrons mainly associated with AMR in clinical samples [14]. Class 1 and 2 integrons were detected in a high percentage of *E. coli* strains that were obtained from humans and animals [15].

The potential sources of eggs contaminated with pathogenic-resistant bacteria have been investigated to a much less extent [16, 17], and no reports have examined the issue in Thailand. However, both pathogenic and non-pathogenic *E. coli* can be a source of drug resistance, thereby playing an important role in the transmission of mobile genetic resistance genes [17, 18]. Therefore, the study aimed to investigate the prevalence of virulence, AMR, and integrons of *E. coli* in cage, free-range, and organic eggs in Phayao Province, Thailand.

## Materials and Methods

### Ethical approval

Ethical approval was exempted in this study. The samples were not related to humans and animals.

### Study period and location

This study was conducted from January to December 2019. The commercial egg samples were purchased from supermarkets in Amphoe Muang, Phayao, Province, Thailand.

### Sample collection

Seven hundred and fifty commercial egg samples from three egg production systems, that is, 250 cage eggs (hens submitted to intensive production housing systems), 250 free-range eggs (hens reared in free-run [barn or aviary] housing systems with access to outdoor runs), and 250 organic eggs (hens raised in free-range housing systems and only provided organic certified feed) were purchased from supermarkets in Phayao Province, Thailand, 2019. Information about the samples was observed from the individual certified labels attached to the egg packages. The egg samples were kept in an icebox and immediately transferred to the microbiology laboratory at School of Medical Sciences, University of Phayao.

### Bacteria isolation and identification

The collected whole eggs were soaked in 10 mL of buffered peptone water (BPW, Difco, Detroit, MI, USA) in a sterile plastic bag at ambient temperature (25°C) for 10 min [7] and then removed with sterile forceps. Bacteria from the eggshells were pre-enriched in BPW broth at 37°C for 18–24 h. After the pre-enrichment incubation, the cultures from the BPW broth of each sample were streaked onto MacConkey agar (MC; Difco, Detroit, MI, USA) and incubated at 37°C for 18–24 h for the isolation step of *E. coli*. The typical colony of *E. coli*, presented as red or pink with a halo zone around it, was picked, streaked, and stabbed onto a triple sugar iron agar (TSI; Difco, Detroit, MI, USA) slant. After the incubation at 37°C for 18–24 h., the presumptive positive reaction of *E. coli* on TSI (lactose fermenter) exhibited yellow with gas production [19]. Then, the TSI culture was grown on trypticase soy agar (TSA; Difco, Detroit, MI, USA) for further confirmed identification by biochemical test using API 20E (bioMérieux Vitek, Inc., Hazelwood, Mo.) according to the manufacturer’s protocols. The identified *E. coli* strains were kept as stocks in 20% glycerol and stored at −80°C until use.

### DNA extraction

*Escherichia coli* strains were sub-cultured in Luria-Bertani broth (LB Broth; Difco, Detroit, MI, USA) at 37°C for 15–18 h, and DNA was extracted using a Qiagen’s QIAamp^®^ DNA mini kit (Qiagen, Hilden, Germany). The extracted DNA of *E. coli* strains was kept at −20°C until use.

### Antimicrobial susceptibility testing

The agar disk diffusion method was carried out according to the guidelines of the Clinical and Laboratory Standards Institute [20]. All *E. coli* strains were tested for eight classes of 15 antimicrobial agents, including aminoglycosides (gentamicin, kanamycin, and streptomycin), phenicols (chloramphenicol), monobactams (imipenem and meropenem), cephalosporin (cefotaxime, ceftazidime, and cefepime), beta-lactam (ampicillin and amoxicillin/clavulanic acid), quinolones (ciprofloxacin and nalidixic acid), folate pathway antagonists (sulfamethoxazole/trimethoprim), and tetracyclines (tetracycline) (Oxoid Ltd., Basingstoke, UK). Mueller-Hinton agar (Oxoid) was used as a culture medium for the assays. For quality control, *E. coli* ATCC 25922 was used as a reference strain. The MDR of *E. coli* was evaluated according to the MDR definition of resistance to one or more agents in three or more antimicrobial classes [21].

### Detection of virulence genes

All *E. coli* strains were evaluated for virulence genes by polymerase chain reaction (PCR) amplification. The primer sequences of the virulence genes were *stx, stx2, eae, bfpA, aggR, elt, esth, estp, invE*, and *astA* and PCR conditions were followed as previously described (Table-1) [22–25]. Amplicons were analyzed by 2% agarose gel electrophoresis, stained with ethidium bromide, and visualized under ultraviolet light using a gel documenting system (BIS 303 PC, Jerusalem, Israel).

**Table-1:** Oligonucleotide primers used for PCR amplification of target genes.

Target gene	Sequence (5’-3’)	Amplicon size (bp)	Reference
*aggR*	GTATACACAAAAGAAGGAAGC	254	[25]
	ACAGAATCGTCAGCATCAGC		
*bfpA*	AATGGTGCTTGCGCTTGCTGC	324	[24]
	GCCGCTTTATCCAACCTGGTA		
*eae*	CCCGAATTCGGCACAAGCATAAGC	881	[23]
	CCCGGATCCGTCTCGCCAGTATTCG		
*astA*	CCATCAACACAGTATATCCG	101	[22]
	ACGGCTTTGTAGTCCTTCCA		
*elt*	AACGTTCCGGAGGTCTTATG	511	[22]
	CAACCTTGTGGTGCATGATC		
*esth*	TTCACCTTTCCCTCAGGATG	172	[22]
	ATAGCACCCGGTACAAGCAG		
*estp*	ACTGAATCACTTGACTCTTCA	120	[22]
	TCACAGCAGTAAATGTGTTGT		
*invE*	GCAGGAGCAGATCTTGAAC	208	[22]
	GAAAGGCACGAGTGACTTTC		
*stx*	AGTTATGTGGTGGCGAAGG	347	[22]
	CACCAGACAATGTAACCGC		
*st×2*	TTCGGTATCCTATTCCCGG	589	[22]
	CGTCATCGTATACACAGGAG		
*Int1*	ACGAGCGCAAGGTTTCGGT	565	[26]
	GAAAGGTCTGGTCATACATG		
*Int2*	GTGCAACGCATTTTGCAGG	403	[26]
	CAACGGAGTCATGCAGATG		

PCR=Polymerase chain reaction

### Detection of classes 1 and 2 integrons

The presence of classes 1 and 2 integrons in all strains was determined by multiplex PCR. The primers are shown in Table-1, and the PCR condition was followed as described previously [26]. The PCR mixture (25 μL) contained 1× buffer solution (Promega, USA), 2 mM MgCl_2_, 0.2 mM dNTP s, 0.4 μM of each primer, 1 U *Taq* polymerase (Promega), and 2 μL of DNA template. Amplicons were analyzed as described above.

### Statistical analysis

The data were analyzed using Statistical Package for the Social Sciences (SPSS) software (version 18.0. SPSS Inc). The prevalence rates of *E. coli* contaminated on the eggshells, AMR, virulence genes, and class 1 and class 2 integrons in each of the three egg production systems were expressed as percentages. Then, the prevalence of *E. coli* contamination on eggshells, their virulence genes, AMR, and class 1 and 2 integrons were compared among the three production systems using the Chi-square test or Fisher’s exact tests. p < 0.05 was considered significant.

## Results

### *Escherichia coli* and virulence genes from eggshells

Out of the 750 eggshell samples from the three production systems, 91 samples (12.1%) were identified as having *E. coli*: 24 strains (26.4%) from cage eggs, 27 strains (29.7%) from free-range eggs, and 40 strains (44%) from organic eggs. There were no significant differences in *E. coli* contamination across the production systems (p > 0.05). Among the *E. coli* strains, 47 (51.6%) strains were positive for at least one tested virulence gene. Table-2 shows the *E. coli* carrying virulence gene strains in each production systems. The highest proportion was detected in organic eggs at 57.5% (23/40), followed by cage eggs with 54.2% (13/24), and free-range eggs with 40.7% (11/27) (p > 0.05). In addition, 13 virulence gene profiles were detected, and the most common virulence gene was *astA* 18.7% (17/91), followed by *bfpA* 9.9% (9/91) and *astA+ bfpA* 6.6% (6/91). Only two virulence genes, *astA* and *bfpA*, were found in all three types of eggshell samples.

**Table-2:** Virulence gene profiles of *Escherichia coli* isolated from eggshells in cage, free-range, and organic eggs.

Virulence Profiles	No. (%) of virulence genes profiles of *Escherichia coli* in different egg productions			Total
	
	Cage	Free-range	Organic	(n=91)

	(n=24)	(n=27)	(n=40)	
Negative	11 (45.8)	16 (59.3)	17 (42.5)	44 (48.4)
Positive virulence	13 (54.2)	11 (40.7)	23 (57.5)	47 (51.6)
*aggR*	0	0	1 (2.5)	1 (1.1)
*astA*	6 (25)	6 (22.2)	5 (12.5)	17 (18.7)
*bfpA*	3 (12.5)	1 (3.7)	5 (12.5)	9 (9.9)
*eae*	0	1 (3.7)	3 (7.5)	4 (4.4)
*stx*	0	0	1 (2.5)	1 (1.1)
*st×2*	1 (4.2)	0	1 (2.5)	2 (2.2)
*aggR+astA*	1 (4.2)	0	0	1 (1.1)
*aggR+eae*	0	0	1 (2.5)	1 (1.1)
*astA+bfpA*	0	1 (1.1)	5 (5.5)	6 (6.6)
*eae+astA*	1 (4.2)	1 (3.7)	0	2 (2.2)
*bfpA+stx*	0	1 (3.7)	0	1 (1.1)
*eae+stx*	0	0	1 (2.5)	1 (1.1)
*astA+bfpA+esth*	1 (4.2)	0	0	1 (1.1)

*aggR*=Aggregative adherence fimbria, *astA*=Enteroaggregative *Escherichia coli* heat-stable enterotoxin, *bfpA*=Bundle-forming pilus A, *eae*=Intimine, *stx*=Shiga toxin, *st×2* = Shiga toxin2, *esth*=Heat stable toxin

### Antimicrobial resistance and MDR

All *E. coli* strains were tested for AMR to 15 different antimicrobial agents by the disk diffusion method. The percentage of *E. coli* strains resistant to one or more antimicrobial agents was 91.2% (83/91). The proportion of *E. coli* resistant to the 15 antimicrobial agents in the eggshell samples was high in cage eggs at 100% (24/24), free-range eggs at 92.6% (25/27), and organic eggs at 85.0% (34/40) (Table-3). *Escherichia coli* resistance to streptomycin, ampicillin, and tetracycline was the most prevalent at 70.3% (64/91), 64.8% (59/91), and 61.5% (56/91), respectively.

**Table-3:** Antimicrobial resistance and integrons of 91 *Escherichia coli* strains isolated from eggshells in cage, free-range, and organic eggs.

Antimicrobial Agents	No. (%) of antimicrobial resistance of *Escherichia coli*				No. (%) of Integrons-positive strains (n = 27)

	Cage (n = 24)	Free-range (n = 27)	Organic (n = 40)	Total (n = 91)	
Aminoglycosides					
GEN	0	1 (3.7)	0	1 (1.1)	0
KAN	9 (37.5)	6 (22.2)	15 (37.5)	30 (33.0)	10 (37.0)
STR	22 (91.7)	19 (70.4)	23 (57.5)	64 (70.3)	22 (81.5)
Phenicols					
CHL	1 (4.2)	3 (11.1)	3 (7.5)	7 (7.7)	3 (11.1)
Monobactams					
IMP	0	0	1 (2.5)	1 (1.1)	0
MEM	9 (37.5)	2 (7.4)	5 (12.5)	16 (17.6)	6 (22.2)
Cephalosporin					
CTX	0	2 (7.4)	0	2 (2.2)	1 (3.7)
CAZ	0	1 (3.7)	0	1 (1.1)	1 (3.7)
CPM	0	3 (11.1)	0	3 (3.3)	1 (3.7)
Beta-lactam					
AMP	15 (62.5)	22 (81.5)	22 (55.0)	59 (64.8)	18 (66.7)
AMC	6 (25.0)	5 (18.5)	11 (27.5)	22 (24.2)	9 (33.3)
Quinolones					
CIP	2 (8.3)	4 (14.8)	0	6 (6.6)	2 (7.4)
NAL	5 (20.8)	9 (33.3)	4 (10.0)	18 (19.8)	9 (33.3)
Folate pathway antagonists					
SXT	7 (29.2)	10 (37.0)	5 (12.5)	22 (24.2)	9 (33.3)
Tetracyclines					
TE	19 (79.2)	21 (77.8)	16 (40.0)	56 (61.5)	18 (66.7)

AMP=Ampicillin, AMC=Amoxicillin/clavulanic acid, CTX=Cefotaxime, CAZ=Ceftazidime, CHL=Chloramphenicol, CIP=Ciprofloxacin, CPM=Cefepime, GEN=Gentamicin, IPM=Imipenem, KAN=Kanamycin, MEM=Meropenem, NAL=Nalidixic acid, STR=Streptomycin, TET=Tetracycline, SXT=Sulfamethoxazole/trimethoprim

Interestingly, a high proportion of *E. coli* strains resistant to streptomycin, ampicillin, and tetracycline was detected in each production system. In addition, 57.1% (52/91) of the *E. coli* strains were MDR. The proportion of MDR strains from cage eggs, 75.0% (18/24), was higher than that from free-range eggs, 63.0% (17/27), and from organic eggs, 42.5% (17/40). There was no statistically significant difference between *E. coli* from the eggshells of the three production systems (p > 0.05). As described in Table-4, 50 AMR profiles were detected. The most common MDR profile was STR-AMP-TET, 5.5% (5/91). Only one *E. coli* strain (1.1%) was resistant to 9 tested antimicrobial agents isolated from organic egg samples.

**Table-4:** Antimicrobial resistance profiles of *Escherichia coli* isolated from eggshells in cage, free-range, and organic eggs.

Antimicrobial resistance profiles	No. (%) of antimicrobial resistance profiles of *Escherichia coli* in different egg productions			Total (n = 91)

	Cage (n = 24)	Free-range (n = 27)	Organic (n = 40)	
MDR	18 (75)	17 (63)	17 (42.5)	52 (57.1)
One drug				
AMP	0	1 (3.7)	3 (7.5)	4 (4.4)
IPM	0	0	1 (2.5)	1 (1.1)
KAN	0	0	2 (5.0)	2 (2.2)
NAL	0	0	1 (2.5)	1 (1.1)
STR	1 (4.2)	0	3 (7.5)	4 (4.4)
Two drugs				
AMC-TET	0	0	1 (2.5)	1 (1.1)
AMP-TET	1 (4.2)	1 (3.7)	1 (2.5)	3 (3.3)
CTX-TET	0	1 (3.7)	0	1 (1.1)
KAN-STR	1 (4.2)	0	1 (2.5)	2 (2.2)
NAL-TET	0	1 (3.7)	0	1 (1.1)
STR-TET	0	0	1 (2.5)	1 (1.1)
Three drugs				
AMP-AMC-TET	0	2 (7.4)	0	2 (2.2)
KAN-AMP-AMC	0	0	1 (2.5)	1 (1.1)
KAN-STR-AMP	0	1 (3.7)	1 (2.5)	2 (2.2)
KAN-STR-MEM	2 (8.3)	0	1 (2.5)	3 (3.3)
KAN-STR-NAL	0	1 (3.7)	0	1 (1.1)
MEM-AMP-AMC	1 (4.2)	0	0	1 (1.1)
STR-AMP-TET	3 (12.5)	0	2 (5)	5 (5.5)
STR-CHL-AMP	0	0	1 (2.5)	1 (1.1)
STR-MEM-AMP	0	0	1 (2.5)	1 (1.1)
STR-MEM-TET	2 (8.3)	0	0	2 (2.2)
STR-NAL-TET	2 (8.3)	0	0	2 (2.2)
STR-SXT-TET	1 (4.2)	0	0	1 (1.1)
Four drugs				
KAN-STR-AMP-TET	1 (4.2)	2 (7.4)	1 (2.5)	4 (4.4)
KAN-STR-MEM-AMP	0	0	1 (2.5)	1 (1.1)
KAN-STR-SXT-TET	0	0	1 (2.5)	1 (1.1)
STR-AMP-AMC-SXT	0	1 (3.7)	0	1 (1.1)
STR-AMP-AMC-TET	1 (4.2)	0	2 (5)	3 (3.3)
STR-AMP-CIP-TET	0	1 (3.7)	0	1 (1.1)
STR-AMP-SXT-TET	0	2 (7.4)	0	2 (2.2)
STR-CHL-AMP-TET	0	0	1 (2.5)	1 (1.1)
Five drugs				
GEN-STR-AMP-SXT-TET	0	1 (3.7)	0	1 (1.1)
KAN-AMP-AMC-SXT-TET	0	0	1 (2.5)	1 (1.1)
KAN-STR-AMP-SXT-TET	1 (4.2)	0	0	1 (1.1)
KAN-STR-MEM-AMP-AMC	0	0	1 (2.5)	1 (1.1)
STR-AMP-AMC-NAL-TET	0	0	1 (2.5)	1 (1.1)
STR-AMP-CIP-NAL-TET	1 (4.2)	1 (3.7)	0	2 (2.2)
STR-AMP-NAL-SXT-TET	0	1 (3.7)	0	1 (1.1)
Six drugs				
KAN-STR-AMP-AMC-NAL-TET	0	0	1 (2.5)	1 (1.1)
KAN-STR-AMP-AMC-SXT-TET	0	0	2 (5.5)	2 (2.2)
KAN-STR-MEM-AMP-SXT-TET	1 (4.2)	1 (3.7)	0	2 (2.2)
STR-AMP-AMC-CIP-NAL-TET	1 (4.2)	0	0	1 (1.1)
STR-AMP-AMC-NAL-SXT-TET	0	1 (3.7)	0	1 (1.1)
STR-CHL-AMP-NAL-SXT-TET	1 (4.2)	2 (7.4)	0	3 (3.3)
STR-CPM-AMP-CIP-NAL-TET	0	1 (3.7)	0	1 (1.1)
STR-MEM-AMP-CIP-NAL-TET	0	1 (3.7)	0	1 (1.1)
Seven drugs				
KAN-STR-CHL-CPM-AMP-SXT-TET	0	1 (3.7)	0	1 (1.1)
KAN-STR-MEM-AMP-AMC-SXT-TET	3 (12.5)	0	0	3 (3.3)
STR-CTX-CAZ-CPM-AMP-AMC-TET	0	1 (3.7)	0	1 (1.1)
Nine drugs				
KAN-STR-CHL-MEM-AMP-AMC-NAL-SXT-TET	0	0	1 (2.5)	1 (1.1)

MDR=Multidrug resistance, The highlight determined MDR of *Escherichia coli* strains, AMP=Ampicillin, IPM=Imipenem, KAN=Kanamycin, NAL=Nalidixic acid, STR=Streptomycin, TET=Tetracycline, SXT=Sulfamethoxazole/trimethoprim, AMC=Amoxicillin/clavulanic acid, MEM=Meropenem, CAZ=Ceftazidime, CHL=Chloramphenicol

### Occurrence of class 1 and 2 integrons

The *E. coli* strains were tested for the presence of class 1 or 2 integrons (*int1* and *int2*) by multiplex PCR amplification, and the results showed 29.7% (27/91) positivity. Among the 27 integron-positive strains, 96.3% (26/27) carried class 1 integron, while 3.7% (1/27) carried class 2 integron (organic eggs). The three production systems had approximately the same proportion of integron-positive strains (cage eggs, 29.2%; free-range eggs, 29.6%; and organic eggs, 30.0%). The highest proportion of streptomycin-resistant strains was positive for integron at 81.5% (22/27), followed by ampicillin-resistant strains, 66.7% (18/27), and tetracycline-resistant strains, 66.7% (18/27). Furthermore, the proportion of MDR strains positive for integron was 34.6% (18/52), and this was similar in all three production systems (cage eggs, 33.3%; free-range and organic eggs, 35.3% each).

### Occurrence of virulence genes and AMR

The coexistence of virulence genes and AMR in *E. coli* strains was analyzed. The results revealed that 53.2% (25/47) of *E. coli* strains carrying virulence genes had MDR. The highest proportion of *E. coli* carrying virulence genes and MDR occurred in cage eggs, 76.9% (10/13), followed by free-range eggs, 63.6% (7/11), and then organic eggs, 34.8% (8/23). Moreover, *E. coli* carrying virulence genes were most commonly detected in streptomycin-resistant strains, 68.1% (32/47), followed by ampicillin-resistant strains, 61.7% (29/47), and then tetracycline-resistant strains, 51.1% (24/47). All *E. coli* carrying virulence genes in the three production systems were susceptible to the cephalosporin class. Moreover, eight strains of *E. coli* carried virulence genes and integrons and showed MDR phenotypes.

## Discussion

Eggshells are exposed to bacterial contamination from different sources such as feces, water, caging material, nesting material, hand, soil, or dust [8, 27], and the presence of *E. coli* in foods indicates direct or indirect contamination with feces or contaminated fecal material [28]. However, this study showed a low incidence of *E. coli* contamination on eggshells from three egg production systems: 16% (40/250) in organic eggs, 10.8% (27/250) in free-range eggs, and 9.6% (24/250) in cage eggs. These results accord with previous studies conducted in Australia (7%, 35/500) [16] and Spain (15.0%, 27/180) [8] but not with studies in which the proportion of *E. coli* contamination was higher, that is, in Spain (25%) [17] and Zambia (34.3%, 74/216) [29]. The different levels of *E. coli* contamination in eggshells depend on many factors, including housing systems, farm management, storage process, and sale (fresh market/supermarket) [8, 30].

The highest proportion of *E. coli* contamination in eggshell samples was detected from organic eggs in this study. This might be due to the production system without the use of antimicrobials and contact with the environment influencing microbiota contamination. Therefore, bacteria isolated from such eggs have more genetic variability than bacteria from eggs produced in a controlled environment [17].

Moreover, *E. coli* from eggshells could act as a vehicle for pathogens causing infection in the food chain [31]. This study revealed that 51.6% (47/91) of *E. coli* strains were positive for at least one tested virulence gene. The highest proportion of *E. coli* strains carrying virulence genes was detected in organic egg samples 57.5% (23/40). In particular, three strains were positive for Shiga toxin genes (*stx* and *stx2*). Furthermore, the *astA* gene encoding for heat-stable enterotoxins was the most common. The presence of *astA* is not restricted to EAEC but is also carried by other pathotypes such as EHEC, ETEC, and EPEC, from both humans and animals [32], and the role of *astA* in the pathogenicity of *E. coli* is still unclear [8].

In addition, this study had a low detection rate of the *esth* gene associated with ETEC strains. A previous report also showed a low detection rate of ETEC strains [16]. Typical EPEC strains are known to have the *eae* gene and *bfpA* gene, while atypical EPEC strains have only the *eae* gene [33]. Both typical and atypical EPEC were detected in this study. Burgos *et al*. [8] reported atypical EPEC commonly found in egg samples in Spain. Moreover, the positive result in the screening for virulence genes raises concerns about the possible transmission of virulent *E. coli* strains through the food chain [8].

Tetracyclines and streptomycin have been commonly used in animal production, and their residues have persisted on animal farms [34]. The results of this study revealed a high level of AMR for streptomycin (70.3%), ampicillin (64.8%), and tetracycline (61.5%) in *E. coli* from the eggshell samples. The results of AMR revealed a higher prevalence compared with the results in Spain [8], Athens [35], and Australia [36]. Moreover, the proportion of AMR in cage eggs was higher than those in free-range and organic eggs, indicating a higher level of antimicrobial use in cage egg production systems [37]. MDR phenotypes have become more common in farm animals and constitute a global health concern [38]. In the present study, 57.1% (52/91) of *E. coli* strains detected in the three egg production systems had MDR, indicating the use of antimicrobials in egg production or co-selection or cross-resistance from other substances [8]. This was a higher MDR prevalence than in South Korea (46.6%) [39], Spain (15.5%) [17], and Australia (22%) [36]. The highest MDR prevalence was found in cage eggs (75%), followed by free-range eggs (63%) and organic eggs (42.5%). The prevalence of *E. coli* MDR has a strong association with antimicrobial use [37]. MDR not only affects animals but also has an impact on the environment by contaminating soil, water, and human bodies through the transfer of resistant organisms through the food chain and/or direct contact with farm personnel [40].

One of the most important factors in the development of resistance to antibiotics is the remarkable ability of bacteria to share genetic resources through lateral gene transfer, including plasmid, transposon, and integrons [41]. Integrons can disseminate antimicrobial-resistant genes by horizontal or vertical transfer and play an important role in the evolution and dissemination of MDR in Gram-negative bacteria [42]. Class 1 integrons of *E. coli* are commonly found in clinical and commensal strains from livestock, companion animals, and exotics [43]. In this study, 29.7% (27/91) of *E. coli* strains were positive for class 1 or 2 integrons, with class 1 integrons occurring at a much higher rate. These findings were similar with those of other studies reporting the predominance of class 1 in human-derived *E. coli* and animal-associated Gram-negative bacteria [14, 44, 45]. Class 2 integrons have been commonly reported in some species of Gram-negative organisms such as *Acinetobacter*, *Enterobacteriaceae*, *Salmonella*, and *Pseudomonas*. However, the prevalence of integron detection in animal farms might be due to the type of animals, farm size, and the amount or frequency of antimicrobials used on the farms [32]. Moreover, the high detection of integrons indicated the possibility of dissemination of antimicrobial-resistant genes between bacteria and potential transfer to humans [45].

Understanding AMR determinants in pathogenic bacteria inhabiting the food ecosystem is key for the assessment of potential risks to consumers [46]. This study showed that *E. coli* strains carrying virulence genes had high resistance to streptomycin, ampicillin, and tetracycline. In the study conducted by Okorie-Kanu *et al*. [47], *E. coli* carrying virulence strains from eggshells had higher resistance to streptomycin and tetracycline than those in this study. A couple of highly antimicrobial-resistant *E. coli* strains carrying virulence genes will cause severe illness, resulting in difficult treatment and increasing morbidity and mortality in animals and humans.

## Conclusion

This is the first report on the prevalence of virulence genes, AMR, and integrons of *E. coli* isolated from eggshells from cage, free-range, and organic eggs sold in supermarkets in Thailand. The findings indicate that eggs from these three types of egg production farms may be potential sources of pathogenic and MDR *E. coli* infection in consumers. It is recommended that the practices of good farm management, good hygiene, and reduction of antimicrobial use should be emphasized on animal farms, especially in egg production farms, to ensure egg safety. In addition, food handlers should be aware of possible cross-contamination of pathogenic and MDR *E. coli* or other pathogenic bacteria from eggshells to other food materials, work surfaces, or utensils when handling or cooking, as a public health concern.

## Authors’ Contributions

AS, CS, WL, and PK: Collected the samples and performed experiments. OS: Drafted and revised the manuscript. AS and WP: Designed the study, data analysis, and drafted and revised the manuscript. All authors have read and approved the final manuscript.

## References

[1] World Health Organization (2019). Salmonella (Non-Typhoidal).

[2] Founou L.L, Founou R.C, Essack S.Y (2016). Antibiotic resistance in the food chain:A developing country perspective. Front. Microbiol.

[3] Office of Agricultural Economics (2022). Egg Balance Measure.

[4] Economou V, Gousia P (2015). Agriculture and food animals as a source of antimicrobial resistant bacteria. Infect. Drug Resist.

[5] Clements A, Young J.C, Constantinou N, Frankel G (2012). Infection strategies of enteric pathogenic *Escherichia coli*. Gut. Microbes.

[6] Kaper J, Nataro J, Mobley H (2004). Pathogenic *Escherichia coli*. Nat. Rev. Microbiol.

[7] Utrarachkij F, Pornraungwong S, Siripanichgon K, Nakajima C, Suzuki Y, Suthienkul O (2012). Possible horizontal transmission of *Salmonella* via reusable egg trays in Thailand. Int. J. Food Microbiol.

[8] Burgos M.J.G, Márquez M.L.F, Pulido R.P, Gálvez A, López R.L (2016). Virulence factors and antimicrobial resistance in *Escherichia coli* strains isolated from hen eggshells. Int. J. Food Microbiol.

[9] Tule A, Hassani U (2017). Colonization with antibiotic-resistant *Ecoli* in commensal fecal flora of newborns. Int. J. Curr. Microbiol. Appl. Sci.

[10] Ketkhao P, Thongratsakul S, Poolperm P, Poolkhet C, Amavisit P (2021). Antimicrobial resistance profiles of *Escherichia coli* from swine farms using different antimicrobials and management systems. Vet. World.

[11] Akullian A, Montgomery J.M, John-Stewart G, Miller S.I, Hayden H.S, Radey M.C, Hager K.R, Verani J.R, Ochieng J.B, Juma J, Katieno J, Fields B, Bigogo G, Audi A, Walson J (2018). Multidrug resistant non-typhoidal *Salmonella* associated with invasive disease in Western Kenya. PLoS Negl. Trop. Dis.

[12] Lerminiaux N.A, Cameron A.D (2019). Horizontal transfer of antibiotic resistance genes in clinical environments. Can. J. Microbiol.

[13] Firoozeh F, Mahluji Z, Khorshidi A, Zibaei M (2019). Molecular characterization of class 1, 2 and 3 integrons in clinical multi-drug resistant *Klebsiella pneumoniae* isolates. Antimicrob. Resist. Infect. Control.

[14] Deng Y, Bao X, Ji L, Chen L, Liu J, Miao J, Yu G (2015). Resistance integrons:Class 1, 2 and 3 integrons. Ann. Clin. Microbiol. Antimicrob.

[15] Kheiri R, Akhtari L (2016). Antimicrobial resistance and integron gene cassette arrays in commensal *Escherichia coli* from human and animal sources in IRI. Gut. Pathog.

[16] Chousalkar K.K, Flynn P, Sutherland M, Roberts J.R, Cheetham B.F (2010). Recovery of *Salmonella* and *Escherichia coli* from commercial eggshells and effect of translucency on bacterial penetration in eggs. Int. J. Food Microbiol.

[17] Fenollar A, Doménech E, Ferrús M.A, Jiménez-Belenguer A (2019). Risk characterization of antibiotic resistance in bacteria isolated from backyard, organic, and regular commercial eggs. J. Food Prot.

[18] Osińska A, Korzeniewska E, Harnisz M, Niestępski S (2017). The prevalence and characterization of antibiotic-resistant and virulent *Escherichia coli* strains in the municipal wastewater system and their environmental fate. Sci. Total Environ.

[19] Ewing W.H (1986). Edwards and Ewing's Identification of Enterobacteriaceae.

[20] Clinical and Laboratory Standards Institute (2018). Antimicrobial Susceptibility Testing Twenty-Third Informational Supplement CLSI M100-S23Clinical and Laboratory Standards Institute, Wayne PA.

[21] Magiorakos A.P, Srinivasan A, Carey R.B, Carmeli Y, Falagas M.E, Giske C.G, Harbarth S, Hindler J.F, Kahlmeter G, Olsson-Liljequist B, Paterson D.L, Rice L.B, Stelling J, Struelens M.J, Vatopoulos A, Weber J.T, Monnet D.L (2012). Multidrug-resistant, extensively drug-resistant and pan drug-resistant bacteria:An international expert proposal for interim standard definitions for acquired resistance. Clin. Microbiol. Infect.

[22] Fujioka M, Otomo Y, Ahsan C.R (2013). A novel single-step multiplex polymerase chain reaction assay for the detection of diarrheagenic *Escherichia coli*. J. Microbiol. Methods.

[23] Oswald E, Schmidt H, Morabito S, Karch H, Marchès O, Caprioli A (2000). Typing of intimin genes in human and animal enterohemorrhagic and enteropathogenic *Escherichia coli*:Characterization of a new intimin variant. Infect. Immun.

[24] Gunzburg S.T, Tornieporth N.G, Riley L.W (1995). Identification of enteropathogenic *Escherichia coli* by PCR-based detection of the bundle-forming pilus gene. J. Clin. Microbiol.

[25] Ratchtrachenchai O.A, Subpasu S, Ito K (1997). Investigation on enteroaggregative *Escherichia coli* infection by multiplex PCR. Bull. Dept. Med. Sci.

[26] Su J, Shi L, Yang L, Xiao Z, Li X, Yamasaki S (2006). Analysis of integrons in clinical isolates of *Escherichia coli* in China during the last six years. FEMS. Microbiol. Lett.

[27] Oviasogie E.F, Ogboghodo B.I, Beshiru A, Omoregie O.B, Ogofure P, Ogofure G.A (2016). The microbial burden load of eggshells from different poultry rearing systems in Ekosodin Village, Edo State, Nigeria. J. Appl. Sci. Environ. Manage.

[28] Dierikx C.M, van der Goot J, van Essen-Zandbergen A, Mevius D.J (2018). Dynamics of cefotaxime resistant *Escherichia coli* in broilers in the first week of life. Vet. Microbiol.

[29] Kapena M.S, Muma J.B, Mubita C.M, Munyeme M (2020). Antimicrobial resistance of *Escherichia coli* and *Salmonella* in raw retail table eggs in Lusaka, Zambia. Vet. World.

[30] Loongyai W, Promphet K, Kangsukul N, Noppha R (2010). Detection of *Salmonella* in eggshell and egg content from different housing systems for laying hens. Int. J. Agric. Biol. Eng.

[31] Mitchell N.M, Johnson J.R, Johnston B, Curtiss R, Mellata M (2015). Zoonotic potential of *Escherichia coli* isolates from retail chicken meat products and eggs. Appl. Environ. Microbiol.

[32] Prapasawat W, Intarapuk A, Chompook P, Nakajima C, Suzuki Y, Suthienkul O (2017). Antimcrobial resistance, intgron, virulence gene, and multilocus sequence typing of *Escherichia coli* isolates from postweaning piglets with and without diarrhea. Southeast Asian J. Trop. Med. Public Health.

[33] Yatsuyanagi J, Saito S, Miyajima Y, Amano K, Enomoto K (2003). Characterization of atypical enteropathogenic *Escherichia coli* strains harboring the *astA* gene that were associated with a waterborne outbreak of diarrhea in Japan. J. Clin. Microbiol.

[34] Graslund S, Bengtsson B.E (2001). Chemicals and biological products used in South-East Asian shrimp farming, and their potential impact on the environment - A review. Sci. Total Environ.

[35] Musgrove M.T, Jones D.R, Northcutt J.K, Cox N.A, Harrison M.A, Fedorka-Cray P.J, Ladely S.R (2006). Antimicrobial resistance in *Salmonella* and *Escherichia coli* isolated from commercial shell eggs. Poul. Sci.

[36] Sodagari H.R, Sahibzada S, Robertson I, Habib I, Wang P (2021). Whole-genome comparative analysis reveals an association between *Salmonella* genomic variation and egg production systems. Front. Vet. Sci.

[37] Barton M.D (2014). Impact of antibiotic use in the swine industry. Curr. Opin. Microbiol.

[38] Zalewska M, Błażejewska A, Czapko A, Popowska M (2021). Antibiotics and antibiotic resistance genes in animal manure-consequences of its application in agriculture. Front. Microbiol.

[39] Adesiyun A, Offiah N, Seepersadsingh N, Rodrigo S, Lashley V, Musai L (2006). Frequency and antimicrobial resistance of enteric bacteria with spoilage potential isolated from table eggs. Food Res. Int.

[40] Ma F, Xu S, Tang Z, Li Z, Zhang L (2021). Use of antimicrobials in food animals and impact of transmission of antimicrobial resistance on humans. Biosaf. Health.

[41] Partridge S.R, Kwong S.M, Firth N, Jensen S.O (2018). Mobile genetic elements associated with antimicrobial resistance. Clin. Microbiol. Rev.

[42] Sabbagh P, Rajabnia M, Maali A, Ferdosi-Shahandashti E (2021). Integron and its role in antimicrobial resistance:A literature review on some bacterial pathogens. Iran. J. Basic Sci.

[43] Rehman M.U, Zhang H, Huang S, Iqbal M.K, Mehmood K, Luo H, Li J (2017). Characteristics of integrons and associated gene cassettes in antibiotic-resistant *Escherichia coli* isolated from free-ranging food animals in China. J. Food Sci.

[44] Park J.H, Kim Y.J, Seo K.H (2018). Spread of multidrug-resistant *Escherichia coli* harboring integron via swine farm wastewater treatment plant. Ecotoxicol. Environ. Saf.

[45] Prapasawat W, Intarapuk A (2021). Prevalence of antimicrobial resistance and integrons in *Escherichia coli* isolated from feces of dairy goats in Nong Chok, Bangkok, Thailand. Vet. Integr. Sci.

[46] Loayza F, Graham J.P, Trueba G (2020). Factors obscuring the role of *Ecoli* from domestic animals in the global antimicrobial resistance crisis:An evidence-based review. Int. J. Environ. Res. Public Health.

[47] Okorie-Kanu O.J, Ezenduka E.V, Okorie-Kanu C.O, Ugwu L.C, Nnamani U.J (2016). Occurrence and antimicrobial resistance of pathogenic *Escherichia coli* and *Salmonella* spp in retail raw table eggs sold for human consumption in Enugu state, Nigeria. Vet. World.

